# Probiotic Foods Are Effective on Weight Loss, Biochemical Parameters, and Intestinal Microbiota in Wistar Albino Rats with Obese Microbiota

**DOI:** 10.1155/2022/4569100

**Published:** 2022-03-03

**Authors:** Nadide Gizem Tarakci, Nihal Zekiye Erdem, Emek Dumen

**Affiliations:** ^1^Department of Nutrition and Dietetics Institute of Health Sciences, Istanbul Medipol University, 34810 Istanbul, Turkey; ^2^Department of Nutrition and Dietetics School of Health Sciences, Istanbul Medipol University, 34083 Istanbul, Turkey; ^3^Department of Food Hygiene and Technology Faculty of Veterinary Medicine, Istanbul University-Cerrahpasa, 34500 Istanbul, Turkey

## Abstract

The positive effects of various probiotic foods on weight control, intestinal microbiota, and biochemical markers have been proven by various studies. However, there is no study on such effects of tarhana and kefir + tarhana consumption, a type of Turkish food rich in *Lactobacillus* spp., *Pediococcus pentosaceus*, *Pediococcus acidilactici*, and *Saccharomyces cerevisiae*. This study aimed to determine the changes caused by regular consumption of kefir and/or tarhana for 6 months on weight gain, intestinal microbiota, and biochemical parameters in Wistar albino rats with obese microbiota. Therefore, thirty-five rats were fed with five different methods of oral gavage (*n* = 7 per group): Normal Diet Control (NDC), High Fat Diet Control (HFDC), 6 mL/kg Kefir + High Fat Diet (Kefir + HFD), 0.2 g/kg Tarhana + High Fat Diet (Tarhana + HFD), and 6 mL/kg Kefir + 0.2 g/kg Tarhana + High Fat Diet (Kefir + Tarhana + HFD). Normality tests were evaluated using the One-Sample Kolmogorov test and Histogram graph. Multiple group comparisons were performed using one-way ANOVA and Tukey's HSD post hoc test, and the statistical significances were indicated by different letters (*p* < 0.05). Comparisons by gender were performed using the independent samples *t*-test. Kefir consumption was more effective on decreasing weight gain. Obese microbiota significantly increased blood glucose level and decreased red blood cell (RBC), hematocrit (HCT), hemoglobin, mean corpuscular hemoglobin (MCH), mean corpuscular hemoglobin concentration (MCHC), platelets (PLT), and white blood cells. RBC and HCT values in Kefir + HFD, PLT value in Tarhana + HFD, and mean corpuscular volume (MCV), MCH, and MCHC values in Kefir + Tarhana + HFD were higher than those of other groups (*p* < 0.05). Kefir + tarhana consumption significantly showed an increase in blood glucose. Kefir and/or tarhana induced the abundance of *Lactobacillus* and blocked the abundances of total coliform bacteria and *Escherichia coli* (*p* < 0.05). We demonstrated that kefir was effective in decreasing weight gain, and all dietary interventions induced positive alterations on biochemical findings and intestinal microbiota.

## 1. Introduction 

One hundred trillion microorganisms live in the microbiota [1]. Ninety percent of human intestinal microbiota consist of *Firmicutes*, *Prevotella genera*, *Bacteroidetes* spp., including *Clostridium*, *Enterococcus*, *Lactobacillus,* and *Ruminococcus* [2].

The relationship between adiposity and microbiota in the regulation of energy homeostasis was first demonstrated by Gordon et al., and Bäckhed et al. They found that the weight and adipose tissue of mice devoid of all microorganisms increased after microbial colonization [3, 4]. This change includes an increase in the *Firmicutes*/*Bacteroidetes* ratio, a change in bacterial composition, and a decrease in density [5, 6]. In studies, it has been determined that the microbiota composition changes in obese depending on the diet [5–7]. One of these dietary components is probiotics.

Obesity also causes changes in biochemical parameters. Red blood cells (RBC), as they oxygenate body tissue, provide necessary blood pressure, create viscosity, and transfer carbon dioxide to lungs; white blood cells (WBC), as they have an effect on the immune system; and platelets (PLT), as their effect may change from protective to harmful, have to be in certain levels [8, 9].

Probiotics are live microorganisms that when administered in adequate amounts confer a health benefit on the host [10], and the health benefits are a result of promotion of a healthy intestinal microbiome that competitively excludes pathogenic bacteria [11, 12] as well as alternative to antibiotics [13, 14]. Recent research showed the importance of probiotics in various medical fields such as dermatology, dentistry, and gastroenterology [15–17]. Also, the probiotic supplementation displayed affirmative impacts on the performance blood hematology and intestinal morphology [18, 19]. Various studies have proved the positive effects of oral intake of various probiotic foods on weight control, antistress, and antitocsin, intestinal microbiota, and biochemical parameters [20–24]. However, there is no study about such effects on health of tarhana with lactic acid bacteria, which is a traditional food mostly consumed as soup in Turkey [25]. We hypothesized that kefir and/or tarhana might induce weight loss, positive alterations of biochemical parameters and intestinal microbiota. Therefore, in this study, we aimed to determine the changes caused by regular consumption of kefir and tarhana for 6 months on weight gain, intestinal microbiota, and biochemical parameters in Wistar albino rats with an obese ecological community.

Experimental animals were preferred due to difficulties in forming and following up experimental groups in humans, due to differences in dietary habits, lifestyle, biochemical values, and chronic diseases. According to the Universal Declaration of Animal Rights, experimental animals that can be used in research are mice and rats. We chose rats, because they are relatively close to human.

## 2. Materials and Methods

### 2.1. Kefir and Tarhana Content

Commercial kefir contained a microbial composition of *Lactobacillus kefiri*, *Lactobacillus kefiranofaciens* subsp. *kefiranofaciens*, *Lactobacillus kefiranofaciens* subsp. *kefirgranum*, *Lactobacillus parakefiri*, *Lactobacillus acidophilus*, *Lactobacillus casei*, *Lactobacillus reuteri*, *Lactobacillus bulgaricus*, *Lactobacillus helveticus*, *Lactobacillus fermentum*, *Lactobacillus plantarum*, *Leuconostoc mesenteroides*, *Streptococcus thermophilus*, *Lactococcus lactis* subsp. *lactis*, *Bifidobacterium bifidum*, *Kluyveromyces marxianus*, and *Saccharomyces cerevisiae* and had a 25.0 × 10^6^ CFU/ml (Kefirzadem®, Isparta, Turkey) [2].

Commercial tarhana contained a microbial composition of *Lactobacillus bulgaricus*, *Lactobacillus plantarum*, *Lactobacillus brevis*, *Lactobacillus casei*, *Pediococcus pentosaceus*, *Pediococcus acidilactici*, and *Saccharomyces cerevisiae*. It had a 10^6^–10^9^ CFU/g (Trakya Ev Tarhanası®, Edirne, Turkey). Tarhana is a traditional fermented food [25].

### 2.2. Animal Experiments

The rats had normal distribution in terms of their initial weight. Thirty-five 8-week-old female and male Wistar albino rats were obtained from the Medipol Medical Research Center (MEDITAM, Istanbul, Turkey). The rats had excellent health. All experiments were conducted under the control of MEDITAM veterinarians in accordance with international ethical rules. The operation was carried out in the center continuously checked by animal welfare units sanctioned by the Ministry of Agriculture and Forestry, and shelter rooms and cages used were in accordance with the legislation. The rats were kept in the room 12 hours light: 12 hours dark throughout the experiment. Environment temperature was between 20 and 24°C. Tecniplast type 2–3 cages with a floor area of 1815 cm^2^ were used. The body weights of the female rats were between 200 and 300 g (mean = 191.80), and the male rats were between 300 and 500 g (mean = 298.07). The male (*n* = 3) and female rats (*n* = 4) in each group were kept in two separate cages to avoid reproduction. Randomization was performed by researchers according to diet types. According to the probiotic foods used in our study, three different probiotic groups were formed. They consumed a high fat diet in the 1^st^ and 2^nd^ months but did not consume kefir and tarhana. The rats were fed with five different methods by oral gavage [*n* = 7 (*n* = 4 female, *n* = 3 male)] and treated as follows: (a) Normal Diet Control (NDC), (b) High Fat Diet Control (HFDC), (c) Kefir + High Fat Diet (Kefir + HFD), (d) Tarhana + High Fat Diet (Tarhana + HFD), and (e) Kefir + Tarhana + High Fat Diet (Kefir + Tarhana + HFD). Healthy microbiota in NDC, obese microbiota in HFDC, Kefir + HFD, Tarhana + HFD, and Kefir + Tarhana + HFD were performed. Therefore, the rats were fed with high fat diet containing 60% fat for 2 months [26]. The same standard feeds were used for all 5 groups. As shown in Table 1, the feeds were produced in the same company, in the same factory, in the same party, and their macronutrient-micronutrient contents were the same (Optima, Party No: 36755, Bolu, August 2019). The initial weights of the rats included in the study were recorded, and when the rats gained 10–25% more weight than their initial weight, they had obese microbiota [27]. After the rats in Kefir + HFD, Tarhana + HFD, and Kefir + Tarhana + HFD had obese intestinal microbiota, they were subjected to daily oral gavages −6 mL of kefir per kilogram of body weight and/or 0.2 g of tarhana per kilogram of body weight, 3 days a week-for 4 months. The rats in the NDC and HFDC did not consume any fermented food or beverage during the study. Kefir and tarhana amounts were calculated based on the recommended consumption amounts in the medical nutrition treatment of individuals (480 ml/day of kefir and 15 g/day of tarhana for 70 kg body weight) [28]. The rats were weighed once a week. The % difference between the weights of the rats with the highest and lightest weights at the beginning of the study was 16%, 14%, 15%, 12%, and 11% in the NDC, HFDC, Kefir + HFD, Tarhana + HFD, and Kefir + Tarhana + HFD groups for female rats, respectively. For male rats, it was 13%, 10%, 16%, 16%, and 15%, respectively. The research was conducted as a single blind study. After the feeding procedures were completed, rats were dispatched. The Implementation Instruction of the Regulation on the Welfare and Protection of Animals Used for Experimental and Other Scientific Purposes, and the Regulation on the Working Procedures and Principles of Animal Experiments Ethics Committees, dated 12/12/2018 and numbered E.3679106, were used. The present animal study was approved by the I.M.U Animal Experiments Local Ethics Committee (38828770–604.01.01-E.46266–04.09.2019). In addition, National Research Council's guide for the care and use of laboratory animals was followed.

### 2.3. Sampling Procedures

Fresh fecal samples were collected on the first day of the study and then once a month. Fecal samples were stored in deep freeze before DNA extraction. Blood samples were collected from the jugular vein on the first day, in the 3^rd^ and 6^th^ months of the study in heparin coated tubes for hematological analysis.

### 2.4. Intestinal Microbiota

After the rats were fed in accordance with the specified procedure, the change of *Lactobacillus* (*L. bacillus*), coliform group, and *Escherichia coli* (*E.coli*) bacterial diversity in the intestines was detected once a month.

*Lactobacillus*: *L. bacillus* were examined by surface plating on De Man, Rogosa and Sharpe (MRS) Agar (Merck, Darmstadt, Germany). The dehydrated broth was heated in distilled water to 68.2 g/l and sterilized at 121°C in a melted autoclave and cooled to 45–50°C. After sterilizing the broth, 10% sorbitol was filtered through a sterile filter with 0.43 *µ*m, and it was added inside to MRS Agar, cooled to 50°C. 1 mL sample of the prepared dilutions was taken into sterile Petri dishes, and 12.5 ml MRS-sorbitol agar was added [29].

Total Coliforms: total coliforms were isolated by surface plating on Violet Red Bile (VRB) Agar (Merck, Darmstadt, Germany). Plates were incubated at 37°C for 24 h [30].

*Escherichia coli*: *E. coli* were examined by surface plating on Tryptone Bile X-glucuronide (TBX) Agar (Merck, Darmstadt, Germany). Plates were incubated at 44°C for 24 h for enumeration [30].

In addition to conventional microbiological cultivation methods, the Polymerase Chain Reaction (PCR) procedure was used for intestinal microbiota analysis. PCR mixture was as follows: 2 µL DNA sample, 2.5 mM MgCl2, 10 mM Tris–HCl pH 8.0, 5 mM KCl (0.2 mM for each nucleotide), 0.8 pmol/mL for each primer, and 1U of Taq DNA polymerase (final 25 lL). The initial denaturation temperature was 94°C for 5 minutes. Then, it was denatured at 94°C for 1 second and afterward 55°C for 1 second for binding of primers and elongated at 72°C for 21 seconds (35 cycles in total). Finally, for the last elongation process, heat treatment at 72°C for 7 minutes was applied to the products, and PCR protocol was completed [31]. Primers designed specifically for the microbiological parameters analyzed in the study to be used in PCR procedures were shown in Table 2 [32, 33].

### 2.5. Blood Parameters

From biochemical parameters fasting plasma glucose level, and from hematological parameters hemoglobin (HGB), hematocrit (HCT), mean corpuscular volume (MCV), mean corpuscular hemoglobin (MCH), mean corpuscular hemoglobin concentration (MCHC), WBC, and PLT were analyzed.

### 2.6. Statistical Analysis

Data were analyzed using Statistical Package for the Social Sciences (SPSS), version 20.0 for Windows (SPSS Inc., Chicago, IL, USA). All the data were presented as mean ± standard deviation (SD). As a result of the literature review, the numbers of animals used in similar studies were taken into consideration [27, 34]. Normality tests were evaluated using the One-Sample Kolmogorov test and Histogram graph. Multiple group comparisons were performed using one-way ANOVA and Tukey's HSD post hoc test, and the statistical significances were indicated by different letters (*p* < 0.05). Comparisons by gender were performed using the independent samples *t*-test. The confidence interval was 95% in all analyses, and the results of intestinal microbiota were assumed to be statistically significant for *p* < 0.05.

## 3. Results

### 3.1. Change in Body Weight

**Figure 1:**
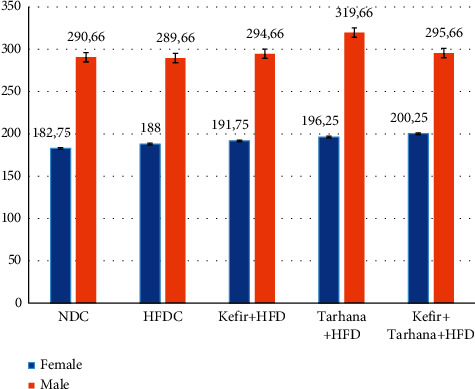
Demographic characteristics of the rats. NDC, Normal Diet Control; HFDC, High Fat Diet Control; Kefir + HFD, Kefir + High Fat Diet; Tarhana + HFD, Tarhana + High Fat Diet; Kefir + Tarhana + HFD, Kefir + Tarhana + High Fat Diet.

**Figure 2:**
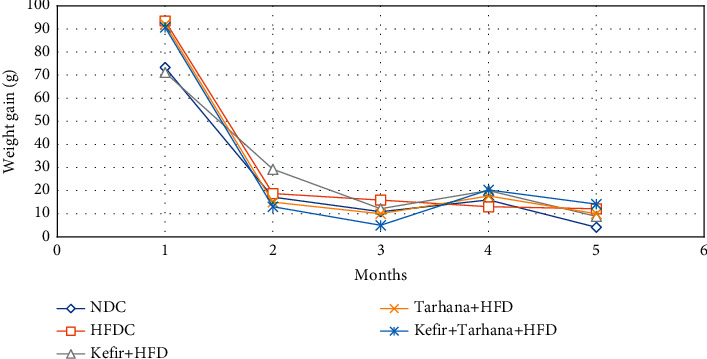
The weight gain before and after dietary intervention by intervention.

**Figure 3:**
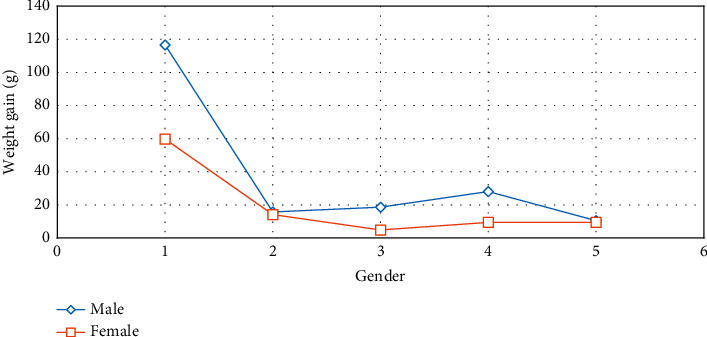
The weight gain before and after dietary intervention by gender.

Before, during, and after the dietary intervention, no adverse event occurred in the health conditions of the rats. Demographic characteristics of the rats were shown in Figure 1. As shown in Table 3 and Figure 2, Kefir + HFD was more effective in reducing weight gain among all interventions. There was a decline in body weight gain as a result of each intervention, especially in the 3^rd^ and 5^th^ months after dietary intervention, but these decreases were not substantial (*p* > 0.05). As shown in Table 3 and Figure 3, it was determined that weight gain slowed down in both genders after dietary intervention, and this increase in females was slower than the males.

### 3.2. Modulation of Intestinal Microbiota Using Kefir and Tarhana

#### 3.2.1. *Lactobacillus*, Total Coliform Group Bacteria, and *E. coli*

As shown in Table 4, consumption of kefir, tarhana, and kefir + tarhana in diets was statistically positively significant with *Lactobacillus*, statistically negatively significant with total coliform group bacteria and *E. coli* abundance parameters. In other words, consumption of kefir, tarhana, and kefir + tarhana in the diets induced the growth of *Lactobacillus* species and blocked the growth of total coliform group bacteria and *E. coli* found in the intestinal flora under normal conditions.

### 3.3. Biochemical Parameters

**Figure 4:**
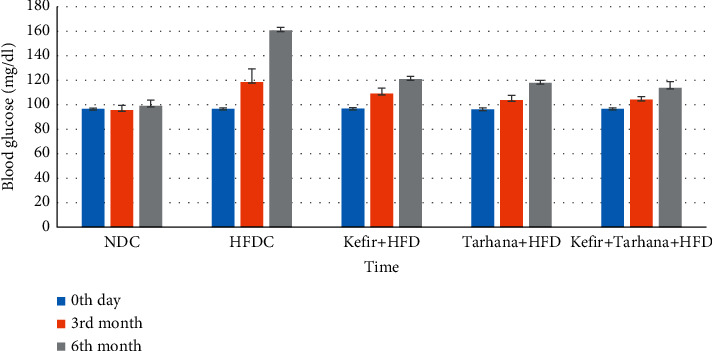
Blood glucose levels of rats before and after dietary intervention.

The biochemical parameters were within the reference value range at the beginning. In the 3^rd^ month, HFDC had the highest blood glucose level (*p* ≤ 0.001). NDC had lower glucose values compared to HFDC and Kefir + HFD, and Kefir + HFD had lower glucose values compared to HFDC (*p* ≤ 0.001). In the 6^th^ month, the blood glucose levels of the Kefir + Tarhana + HFD group were found to be lower than the other groups, and it was statistically significant (*p* ≤ 0.001). As shown in Table 5 and Figure 4, it was determined that Kefir + Tarhana + HFD consumption decreased the blood glucose statistically significantly; but when consumed one by one, the decreases were found to be less (*p* ≤ 0.001).

Based on the results from Table 6, RBC and HCT in Kefir + HFD group; PLT in Tarhana + HFD group; MCV, MCH, and MCHC in Kefir + Tarhana + HFD group were found to be higher than those of other groups. It was determined that Kefir + HFD increased RBC (*p* ≤ 0.001), HCT (*p* ≤ 0.001), HGB (*p* ≤ 0.001), and MCH (*p* ≤ 0.001) levels more than those of other groups. It was concluded that Tarhana + HFD increased PLT (*p* ≤ 0.001) and WBC (*p* ≤ 0.001) levels more than those of other groups. As shown in Table 6, it was determined that Kefir + Tarhana + HFD increased MCV (*p*=0.08) and MCHC (*p* ≤ 0.001) levels more than those of other groups.

## 4. Discussion

In this study, it was determined that fermented products consumed for 6 months in Wistar albino rats with obese microbiota caused weight loss and produced positive changes in biochemical findings and intestinal microbiota. It was determined that kefir and/or tarhana decreased weight gain and blood glucose and positively affected hematological parameters and intestinal microbiota. In recent studies, it has been shown that intestinal microbiota had positive effects on health and disease in vertebrates. These positive effects were also detected in kefir's influence on the reduction in weight gain in our study [27, 35, 36].

Guidelines recommend reducing energy intake, increasing physical activity, and changing lifestyle and nutrition-behavior in the treatment of obesity and obesity-related diseases. Patients and researchers are seeking alternative treatment methods. To this end, other alternative treatment methods applied by obese individuals are nutritional supplements, medicinal plants, herbal mixtures, herbal products, yoga, acupuncture, acupressure, homeopathy, and hypnotherapy [37].

In another study using saline solution and fermented yogurt, it was determined that consumption of yogurt fermented by *L. bacillus plantarum* Q180 decreased weight gain; in another study using milk powder and traditional kefir [38], it was determined that kefir ranks higher in preventing weight gain [39]. The results of these studies were similar to the effects of kefir on weight gain. In another study comparing commercial and traditional kefir, commercial kefir caused more weight gain in rats than traditional [40]. It can be said that this positive effect of kefir on weight gain occurs as a result of some biochemical mechanisms. These include inhibiting 3T3-L1 adipocyte differentiation through downregulation of adipogenic transcription factor expression, suppressing the lipogenesis pathway, inducing Janus kinase 2 (JAK2) signaling through the JAK2/signal transducer and activator of transcription 3 (STAT3) and JAK2/activated protein kinase (AMPK) pathways [39].

In our study, it was found that *L. bacillus* spp. levels were significantly lower in the HFDC compared to Kefir + HFD, Tarhana + HFD, and Kefir + Tarhana + HFD groups (*p* < 0.05). In other words, consumption of kefir and/or tarhana induced the growth of *L. bacillus* spp. and blocked the growth of total coliform and *E. coli* spp. in the intestinal flora. Based on this positive change in intestinal microbiota, it can be stated that kefir and tarhana were consumed in the correct amount and form, had the correct probiotic bacteria content, and were stored and prepared under appropriate conditions. In other words, the study reports that the consumption of kefir and/or tarhana by inhibiting the release of taurine conjugated bile acids prevents the increase of proinflammatory bacteria species. In another study similar to ours, it was reported that the consumption of Tibetan Kefir increased *E. coli* spp. and *Bacteroides* spp. populations [41]. Also, in another study by Tu et al. [42], it was determined that kefir peptides significantly improved the intestinal microbiota although they did not affect the *Firmicutes*/*Bacteroidetes* ratio, known as the obesity biomarker. The common point of all these studies and our study is that the results obtained prevent weight gain and have a positive effect on microbiota and hematological parameters.

In a meta-analysis study conducted in in vitro and animal studies, kefir consumption has been shown to have antioxidative, anticarcinogenic, and antihypertensive effects as well as lowering glucose and cholesterol levels [36]. In other studies, it was determined that consumption of kefir, high protein fermented whey beverage, and pectinase treated probiotic banana juice significantly decreased blood glucose levels [43–45]. The positive effects of fermented foods on blood glucose in these studies were similar to the findings of our study. It is thought that the positive effects of kefir consumption on blood glucose develop due to the antidiabetic properties of *L. bacillus* and *Bifidobacterium*. These features are as follows: they stimulate the uptake of glucose by the muscles to produce insulinotropic polypeptides and glucagon-like peptide 1; induce the storage of more blood glucose in the form of glycogen in the liver; decrease glucose absorption from the gastrointestinal system, and alter the metabolic pathways of glucose [43].

In our study, it was determined that obese microbiota caused a significant decrease in the hematological parameters of RBC, HCT, HGB, MCH, MCHC, PLT, and WBC. Also, it was found that kefir significantly increased RBC, HGB, and MCHC; tarhana significantly increased MCHC and PLT; kefir + tarhana significantly increased HGB, MCHC, and MCH. It has been determined that the HGB values of those who consume kefir, which is a dairy product, increased more. Based on the data of our study, it can be said that kefir and tarhana consumption can provide blood glucose remission, facilitate oxygen transport to cells, and balance blood pressure, and tarhana can support the immune system. The positive findings obtained from another study by Ray et al. [46], in which fermented rice based beverages kept the HGB level in the reference range, are similar to the findings of our study. In another study by Nurliyani et al. [47], kefir consumption was not found to be effective on RBC, HGB, HCT, MCHC, and WBC. When the literature is reviewed on the effects of kefir and/or tarhana consumption on red and white blood cells, platelets in obese individuals, it is seen that more studies should be conducted. These different findings among current studies are thought to be due to differences between fermented food types and consumption amounts, animal subjects, and conditions.

In this study, the effects of kefir and/or tarhana consumption on weight gain, biochemical findings, and intestinal microbiota were demonstrated in Wistar albino rats with obese microbiota. It has been determined that kefir was more effective in preventing weight gain, and kefir and/or tarhana improved biochemical findings and the intestinal microbiota by reducing the density of pathogenic bacteria and increasing the density of beneficial bacteria, changing from dysbiosis to normobiosis.

There are some limitations to our study: first, since there are no studies in the literature regarding the effects of tarhana consumption on weight gain, biochemical findings, and intestinal microbiota, it could not be compared with other studies, and hence, the data of our study were shared in this study. We think that our research will shed light on the researches to be done in this area. Second, only commercial kefir and tarhana were included in the study. In other studies, to be carried out in the future, the comparison of the effects of the commercial kefir-tarhana pair with the traditional kefir-tarhana pair in concordance with the parameters in our study will shed light on the science.

Finally, randomized controlled studies are needed to quantify these effects of fermented foods. The methodology of this study was designed to be applicable to humans. Therefore, considering this methodology, randomized controlled studies on humans will be needed in the future.

As a result, it was determined that kefir consumption was effective in decreasing the weight gain, and kefir + tarhana consumption was effective in decreasing blood glucose. It was determined that kefir increased the hematological parameters of RBC, HCT, HGB, and MCHC, tarhana increased PLT and WBC, and kefir + tarhana increased MCV and MCHC. In addition, it was determined that kefir and/or tarhana consumption prevented the weight gain by inducing the *L. bacillus* spp. population and blocking the total coliform and *E. coli* spp. population.

This experimental evidence shows that fermented products control weight gain and improve gut microbiota and hematological parameters. In addition, it shows that fermented products can improve the metabolic syndrome by positively changing the microbiota in obese individuals. Therefore, it is thought that it may create a new opportunity in the prevention and treatment of diseases associated with obesity.

## Figures and Tables

**Table 1:** Ingredients of Optima whole rat feed.

Additives	IU/kg
Vitamins	
3a672a Vitamin A	36000
E671 Vitamin D3	6500
Analytical components	%
Raw protein	25
Raw cellulose	3
Raw oil	9.01
Raw ash	5.8
Sodium	0.23
Calcium	0.65
Phosphorus	0.55
Lysine	1.42
Methionine	0.63
Trace elements	mg/kg
3b202 Iodine (calcium iodate anhydride	0.8
3b302 Cobalt (cobalt II carbonate)	0.15
E4 Copper	10
3b 502 Manganese (manganese oxide)	50
3b 603 Zinc (zinc oxide)	50
E8 Selenium (sodium selenite)	0.15

Raw materials. Soybean meal (derived from genetically modified soybean), Bonkalite, Corn (derived from genetically modified corn), Rice bran , Soy oil (derived from genetically modified soy), Sugar beet molasses, Calcium carbonate, Lignosulphonate (E 565), Vitamin-Mineral Blend, Sodium chloride, Bentonite (1m558), Calcium Propionate (E282), Yeast (Saccharomyces cerevisiae CNM, I-1077 (4b1711).

**Table 2:** Specific primer sets used in the study.

Primer no.	Sequence (5′–3′)	Target gene/Amp (bp)	Target microorganism
1	AAGAAACTTTGTTTAGTTTTGAGGTA	16s rRNA/227	*Lactobacillus acidophilus*
2	CAATTTTCGTGTCCCCTTCG GTTAATGATAGTGTGTCGAAAC	23S/450	*Escherichia coli*
3	AAGAACTTTGTTCAGTTTTGAGAGTA	16s rRNA/232	*Lactobacillus delbrueckii*, *subs bulgaricus*
4	TTGAAACAATGTTCAGTTTTGAGGGGC	16s rRNA/162	*Lactobacillus brevis*
5	GTAGGTGGCAAGCGTTACC	16s rRNA/228	*Staphylococcus aureus*

**Table 3:** The comparison of weight gain before and after dietary intervention by intervention and gender.

Dietary intervention (*n* = 7 per group)	Body weight gain (g) (M ± SD)				
	Before intervention		After intervention		
	1st month	2nd month	3rd month	4th month	5th month
NDC^a^	73.29 ± 31.31	17.14 ± 9.51	10.86 ± 15.97	16.00 ± 9.66	4.14 ± 7.76
HFDC^b^	93.43 ± 27.43	18.71 ± 12.92	15.86 ± 13.55	13.00 ± 13.18	12.14 ± 11.20
Kefir + HFD^c^	71.14 ± 40.33	29.28 ± 18.62	12.14 ± 10.73	20.00 ± 8.00	8.86 ± 10.43
Tarhana + HFD^d^	92.00 ± 46.88	15.00 ± 8.41	10.00 ± 2.16	17.71 ± 13.92	10.00 ± 8.81
Kefir + Tarhana + HFD^e^	90.71 ± 31.45	13.00 ± 19.82	5.00 ± 6.58	20.29 ± 15.91	14.14 ± 10.70
*p* value	0.66	0.28	0.47	0.80	0.40

Gender					
Male (*n* = 15)	116.60 ± 25.55	15.73 ± 23.97	18.67 ± 9.20	28.07 ± 10.10	10.47 ± 12.16
Female (*n* = 20)	59.75 ± 17.44	14.15 ± 14.17	4.85 ± 8.02	9.40 ± 5.18	9.40 ± 8.08
*p* value^*∗*^	0.00	0.82	0.00	0.00	0.77

NDC, Normal diet control; HFDC, High fat diet control; Kefir + HFD, Kefir + High fat diet; Tarhana + HFD, Tarhana + High fat diet; Kefir + Tarhana + HFD, Kefir + Tarhana + High fat diet. a, b, c, d, e: Lettering represent groups. It states that there is a significant difference between the groups represented by the letters in the lettering (*p* < 0.05). Multiple group comparisons were performed using one-way ANOVA and Tukey's HSD posthoc test. Comparisons by gender were performed using the independent samples *t*-test. *p* values are for changes between groups by dietary intervention. ^*∗*^*p* values are for changes between groups by gender.

**Table 4:** The correlation of intestinal microbiota by dietary intervention.

*Lactobacillus* (*n* = 7 per group)		NDC (*n* = 7)	HFDC (*n* = 7)	Kefir + HFD (*n* = 7)	Tarhana + HFD (*n* = 7)	Kefir + Tarhana + HFD (*n* = 7)
NDC	Correlation coefficient	1.000	0.088	0.−072	0.590	0.509
	Sig. (two-tailed)	—	0.099	0.000	0.001	0.004

HFDC	Correlation coefficient	0.088	1.000	0.777	0.256	0.905
	Sig. (two-tailed)	0.099	—	0.003	0.000	0.001

Kefir + HFD	Correlation coefficient	0.−072	0.777	1.000	0.854	0.609
	Sig. (two-tailed)	0.000	0.003	—	0.063	0.291

Tarhana + HFD	Correlation coefficient	0.590	0.256	0.854	1.000	0.343
	Sig. (two-tailed)	0.001	0.000	0.063	—	0.129

Kefir + Tarhana + HFD	Correlation coefficient	0.509	0.905	0.609	0.343	1.000
	Sig. (two-tailed)	0.004	0.001	0.291	0.129	—

Total coliform group bacteria (*n* = 7 per group)						
NDC	Correlation coefficient	1.000	0.453	0.276	0.118	0.391
	Sig. (two-tailed)	—	0.032	0.002	0.001	0.003

HFDC	Correlation coefficient	0.453	1.000	0.464	0.106	0.045
	Sig. (two-tailed)	0.032	—	0.001	0.002	0.000

Kefir + HFD	Correlation coefficient	0.276	0.464	1.000	0.−084	0.044
	Sig. (two-tailed)	0.002	0.001	—	0.587	0.161

Tarhana + HFD	Correlation coefficient	0.118	0.106	0.−084	1.000	0.367
	Sig. (two-tailed)	0.001	0.002	0.587	—	0.175

Kefir + Tarhana + HFD	Correlation coefficient	0.391	0.045	0.044	0.367	1.000
	Sig. (two-tailed)	0.003	0.000	0.161	0.175	—

NDC, Normal diet control; HFDC, High fat diet Control; Kefir + HFD, Kefir + High fat diet; Tarhana + HFD, Tarhana + High fat diet; Kefir + Tarhana + HFD, Kefir + Tarhana + High fat diet. Numbers written in bold characters are statistically significant (*p* < 0.05).

**Table 5:** The comparison of blood glucose levels before and after dietary intervention.

Dietary intervention (n=7 per group)	Comparison between groups			
	Blood glucose level (78–155 mg/dl) (M ± SD)			
	0^th^ day	3^rd^ month	6^th^ month	*p* value^*∗*^
NDC^a^	96.57 ± 0.53^a^	95.57 ± 3.95^a^	99.14 ± 4.53^a^	0.22
HFDC^b^	96.71 ± 0.76^a^	118.57 ± 10.69b^a,b^	160.57 ± 2.64^ba,bc,c^	0.00
Kefir + HFD^c^	96.85 ± 0.69^a^	109.00 ± 4.47^ca,cb,b^	120.86 ± 2.19^ca,ce,c^	0.00
Tarhana + HFD^d^	96.14 ± 1.34^a^	103.71 ± 3.82^db,b^	117.86 ± 2.04^da,db,c^	0.00
Kefir + Tarhana + HFD^e^	96.71 ± 0.75^a^	104.14 ± 2.41^eb,b^	113.85 ± 4.98^ea,eb,c^	0.00
*p* value	0.59	0.00	0.00	

NDC, Normal diet control; HFDC, High fat diet control; Kefir + HFD, Kefir + High fat diet; Tarhana + HFD, Tarhana + High fat diet; Kefir + Tarhana + HFD, Kefir + Tarhana + High fat diet. a, b, c, d, e: Lettering represent groups. It states that there is a substantial difference among interventions represented by the letters in the double lettering. *p* values are for changes between groups (*p* < 0.05). Multiple group comparisons were performed using one-way ANOVA and Tukey's HSD posthoc test. a, b, c: Lettering represents time points. The difference in averages was made by Bonferroni multiple comparisons. The differences with 95% confidence were marked with different notations. ^*∗*^*p* values are for changes within groups (*p* < 0.05).

**Table 6:** The comparison of hematological parameters before and after dietary.

Hematological parameters	Pre-post	NDC^a^ (*n* = 7)	HFDC^b^ (*n* = 7)	Kefir + HFD^c^ (*n* = 7)	Tarhana + HFD^d^ (*n* = 7)	Kefir + Tarhana + HFD^e^ (*n* = 7)	*p* value
RBC (7.27–9.65 X miilion/mm^3^)	6th month	8.06 ± 1.12^a^	5.72 ± 1.88^ba,b,a^	6.90 ± 1.89^b^	6.13 ± 1.57^b^	5.44 ± 1.34^ea,b^	0.00
	3rd month	8.23 ± 0.90^a^	5.12 ± 0.95^ba,b^	5.02 ± 0.79^ca,b^	5.25 ± 0.98^da,b^	4.61 ± 0.63^ea,b^	0.00
	0th day	8.58 ± 1.42^a^	8.53 ± 1.25^a^	7.63 ± 1.14^a^	8.35 ± 1.50^a^	8.66 ± 1.06^a^	0.59
	*p* value^*∗*^	0.72	0.00	0.02	0.02	0.00	

HCT (39–53 %)	6th month	44.00 ± 0.81^a^	30.85 ± 11.59^b^	34.85 ± 9.90^b,a^	32.71 ± 10.19^b,a^	30.14 ± 7.60^ea,b^	0.04
	3rd month	44.69 ± 0.73^a^	24.31 ± 0.98^ba,b^	26.77 ± 2.70^ca, b^	27.93 ± 3.26^da,b^	25.78 ± 3.04^ea,b^	0.00
	0th day	44.27 ± 3.14^a^	45.97 ± 1.96^a^	45.17 ± 4.43^a^	44.25 ± 3.52^a^	47.80 ± 4.28^a^	0.33
	*p* value^*∗*^	0.24	0.00	0.00	0.00	0.00	

MCV (49-58 um^3^)	6th month	53.57 ± 6.34a	52.28 ± 4.43^b,a^	50.85 ± 5.58^b,a^	52.57 ± 4.92^a^	55.14 ± 4.84^a^	0.66
	3rd month	53.57 ± 0.78^a^	51.42 ± 0.97^ba,b^	51.42 ± 0.97^ca,b^	51.71 ± 0.75^da,a^	50.78 ± 0.56^ea,a^	0.00
	0th day	53.28 ± 1.60^a^	53.75 ± 1.29^a^	54.56 ± 2.14^a^	53.43 ± 1.58^a^	52.98 ± 2.42^a^	0.57
	*p* value^*∗*^	0.85	0.04	0.05	0.25	0.08	

HGB (13.7–17.6 g/dl)	6th month	16.28 ± 0.75a	9.72 ± 3.57^ba,b^	10.98 ± 3.78^ca,b^	10.41 ± 3.44^da,a^	10.22 ± 2.43^ea,c^	0.00
	3rd month	16.49 ± 0.75a	7.25 ± 1.05^ba,b^	7.37 ± 1.36^ca,b^	7.81 ± 1.52^da,a^	7.57 ± 1.41^ea,b^	0.00
	0th day	15.91 ± 0.60a	15.63 ± 1.08^a^	16.48 ± 0.48^a^	16.69 ± 0.66^a^	15.90 ± 0.63^a^	0.05
	*p* value^*∗*^	0.44	0.00	0.00	0.25	0.00	

MCH (17–20 pg)	6th month	16.85 ± 2.26^a^	16.85 ± 2.91^b, a^	16.00 ± 2.08^b, a^	16.57 ± 2.14^b, a^	19.14 ± 1.57^a^	0.11
	3rd month	17.71 ± 0.75^a^	15.42 ± 0.53^ba, b^	15.71 ± 0.48^ca, b^	15.42 ± 0.53^da,b^	15.71 ± 0.75^ea,b^	0.00
	0th day	18.26 ± 1.16^a^	18.20 ± 0.76^a^	18.22 ± 0.76^a^	17.77 ± 0.30^a^	18.54 ± 1.06^a^	0.59
	*p* value^*∗*^	0.50	0.00	0.00	0.00	0.00	

MCHC (33–38 g/dl)	6th month	36.28 ± 0.48^a^	32.00 ± 3.26^ba,b^	31.14 ± 1.57^ca,c^	31.14 ± 1.51^da,b^	33.85 ± 2.34^c^	0.00
	3rd month	36.41 ± 0.56^a^	28.00 ± 2.94^ba,b^	27.00 ± 1.29^ca,b^	28.71 ± 1.49^da,b^	27.57 ± 2.29^ea,b^	0.00
	0th day	36.82 ± 1.27^a^	37.05 ± 1.18^a^	36.62 ± 1.31^a^	36.91 ± 1.10^a^	37.34 ± 0.97^a^	0.83
	*p* value^*∗*^	0.34	0.00	0.00	0.00	0.00	

PLT (638–1117X bin/mm3)	6th month	643.85 ± 0.89a	142.42 ± 15.43^ba,b^	138.85 ± 19.33^ca,b^	147.14 ± 18.09^da,b^	139.71 ± 17.00^ea,b^	0.00
	3rd month	643.28 ± 0.48^a^	133.57 ± 9.30^ba,b^	134.28 ± 5.67^ca,b^	129.08 ± 7.04^da,b^	132.71 ± 9.39^ea,b^	0.00
	0th day	643.02 ± 3.06^a^	641.67 ± 3.51^a^	640.30 ± 2.47^a^	642.28 ± 2.6 9^a^	644.00 ± 2.08^a^	0.17
	*p* value^*∗*^	0.26	0.00	0.00	0.00	0.00	

WBC (2.0–8.25X bin/mm3)	6th month	5.52 ± 0.17^a^	0.58 ± 0.13^ba,b^	0.68 ± 0.23^ca,b^	0.68 ± 0.30^da,b^	0.60 ± 0.23^ea,b^	0.00
	3rd month	5.38 ± 0.18^a^	0.35 ± 0.10^ba,b^	0.39 ± 0.08^ca,b^	0.36 ± 0.10^da,b^	0.36 ± 0.09^ea,b^	0.00
	0th day	5.34 ± 0.21^a^	5.31 ± 0.23^a^	5.46 ± 0.28 a	5.30 ± 0.29^a^	5.29 ± 0.315^a^	0.77
	*p* value^*∗*^	0.17	0.00	0.00	0.00	0.00	

NDC, Normal Diet Control; HFDC, High fat diet control; Kefir + HFD, Kefir + High fat diet; Tarhana + HFD, Tarhana + High fat diet; Kefir + Tarhana + HFD, Kefir + Tarhana + High Fat Diet, a, b, c, d, e: Lettering represents groups. It states that there is a significant difference between the groups represented b y the letters in the double lettering. *p* values are for changes between groups (*p* < 0.05). Multiple group comparisons were performed using one-way ANOVA and Tukey's HSD posthoc test. a, b, c: Lettering represents time points. The difference in averages was made by Bonferroni multiple comparisons. The differ ences with 95% confidence were marked with different notations. ^*∗*^*p* values are for changes within groups (*p* < 0.05).

## Data Availability

The data used to support the findings of this study are available from the corresponding author upon request.
